# Rare Disease Registries Are Key to Evidence-Based Personalized Medicine: Highlighting the European Experience

**DOI:** 10.3389/fendo.2022.832063

**Published:** 2022-03-04

**Authors:** Stefan Kölker, Florian Gleich, Ulrike Mütze, Thomas Opladen

**Affiliations:** Division of Child Neurology and Metabolic Medicine, Center for Child and Adolescent Medicine, Heidelberg University Hospital, Heidelberg, Germany

**Keywords:** rare disease (RD), inherited metabolic diseases, FAIR (findable, accessible, interoperable, and reusable) principles, personalized medicine, patient registry

## Abstract

Rare diseases, such as inherited metabolic diseases, have been identified as a health priority within the European Union more than 20 years ago and have become an integral part of EU health programs and European Reference Networks. Having the potential to pool data, to achieve sufficient sample size, to overcome the knowledge gap on rare diseases and to foster epidemiological and clinical research, patient registries are recognized as key instruments to evidence-based medicine for individuals with rare diseases. Patient registries can be used for multiple purposes, such as (1) describing the natural history and phenotypic diversity of rare diseases, (2) improving case definition and indication to treat, (3) identifying strategies for risk stratification and early prediction of disease severity (4), evaluating the impact of preventive, diagnostic, and therapeutic strategies on individual health, health economics, and the society, and (5) informing guideline development and policy makers. In contrast to clinical trials, patient registries aim to gather real-world evidence and to achieve generalizable results based on patient cohorts with a broad phenotypic spectrum. In order to develop a consistent and sustained framework for rare disease registries, uniform core principles have been formulated and have been formalized through the European Rare Disease Registration Infrastructure. Adherence to these core principles and compliance with the European general data protection regulations ensures that data collected and stored in patient registries can be exchanged and pooled in a protected environment. To illustrate the benefits and limitations of patient registries on rare disease research this review focuses on inherited metabolic diseases.

## Introduction

Any disease affecting less than 1 in 2,000 people is considered rare in the European Union (EU). Although the approximately 6,000-8,000 distinct rare diseases are highly heterogeneous regarding etiology, pathophysiology, clinical presentation, and treatability, they have in common that affected individuals are often confronted with similar problems, such as diagnostic odyssey, lack of safe and effective therapies, and scarce specialist centers, resulting in significant inequalities. On the European level, rare diseases were identified by the 2^nd^ EU Health Program as a healthcare priority with unmet needs, highlighting patient registries as the best way to pool data in order to achieve sufficient sample sizes for epidemiological and clinical research (1).

Rare diseases such as inherited metabolic diseases have been traditionally regarded as Mendelian traits that are caused by specific pathogenic gene variations, and as experiments of nature which helped to decipher underlying mechanisms of monogenic disorders (2). Natural history studies on inherited metabolic diseases, however, often failed to identify clear-cut genotype-phenotype correlations and to predict the clinical phenotype based on the genotype or biochemical phenotype. This is partially explained by involvement of other pathways whose fluxes are changed though secondary inhibition induced by toxic metabolites or lack of substrates (3, 4). Furthermore, modifier genes (5, 6), alternative pathways (7–9), side reactions of enzymes (10), metabolic proof-reading (11), and the concomitant net effect of metabolic changes on the level of single cells, organs and the whole organism significantly contribute to the resulting phenotype. Besides these intrinsic factors, it has been increasingly recognized that metabolism can also be influenced by gene-environment and gene-nutrient interactions (12–14), and the microbiome (15, 16). As a consequence, inherited metabolic diseases are nowadays perceived as a model for complex diseases. Another important aspect that causes apparent phenotypic diversity is time, the fourth dimension setting the scene for ontogeny and ageing. Therefore, the phenotype of an individual at a certain time point of life should not be regarded as static but as a snapshot. Any phenotypic comparisons should include the individual age as an anchor point, the clinical phenotype itself being a complex moving target.

For rare diseases patient registries are thought to be key instruments to achieve a sufficient sample size for clinical research, to guide healthcare planning, and to foster the development and evaluation of diagnostic and therapeutic interventions. Patient registries significantly contribute to evidence-based personalized medicine in the field of rare diseases, since they can be used for multiple purposes, such as improvement of case definition, revision of disease classification, evaluation of indication to treat and risk stratification, as well as safety, effectiveness, feasibility, limitations and benefits of diagnostic and therapeutic strategies under real-world conditions.

## The Evolution of Rare Disease Registries

### From Single Diseases to European Reference Networks (ERN) Registries

**Figure 1:**
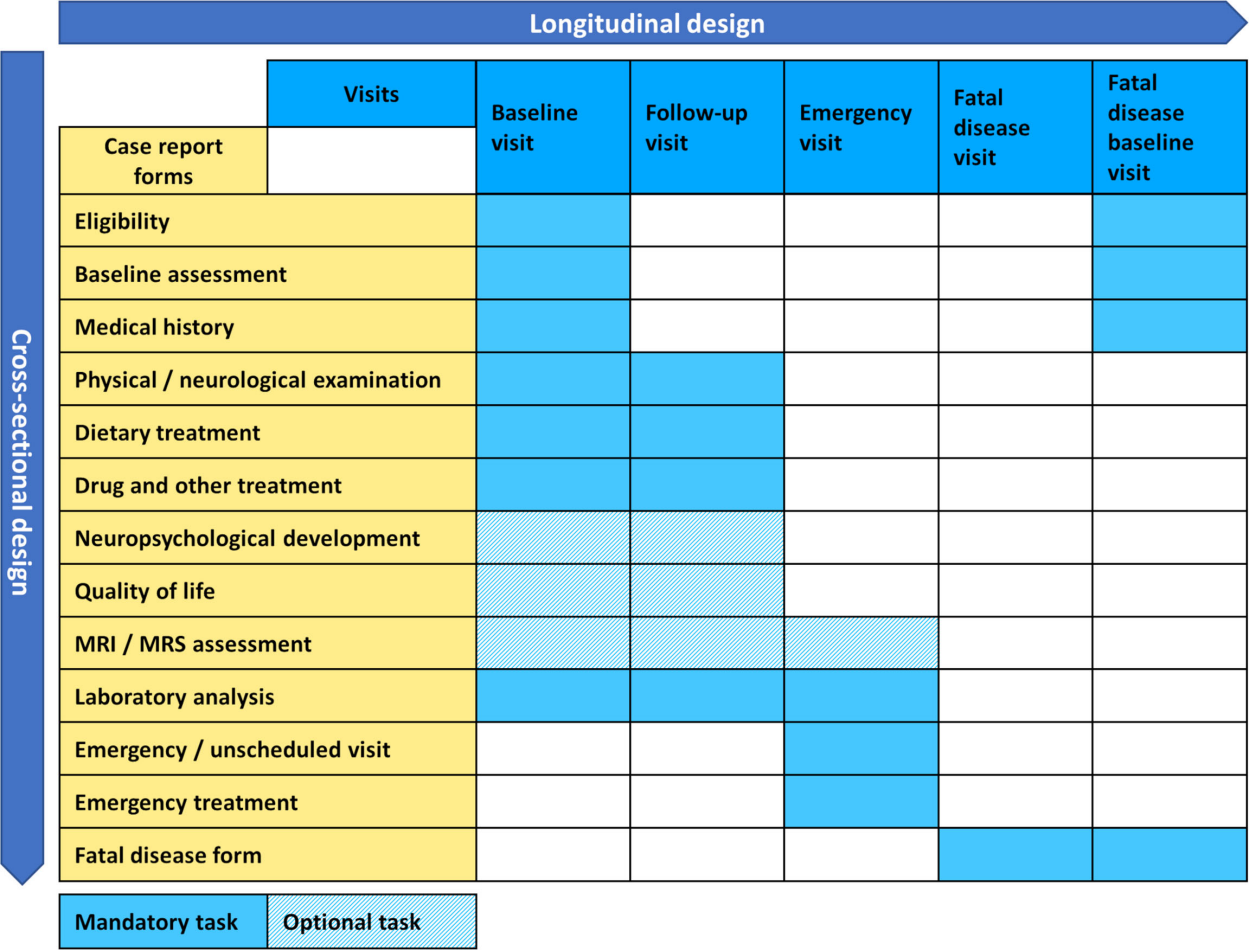
General design of the E-IMD, E-HOD, iNTD and U-IMD registries.

With the benefits of patient registries being widely accepted, the field of rare diseases saw a multitude of individual efforts on registry building in the past, usually realized within a national framework and focusing on single diseases or tightly defined disease groups. Successful examples on a national level include the mitoREGISTRY of the German Network for Mitochondrial Disorders (mitoNET, https://www.mitonet.org/) as well as Leukonet (https://www.leukodystrophie-info.de/), the German Network for Leukodystrophies, gathering data on mitochondrial disease since 2009 and leukodystrophies since 2003 respectively, both receiving funding from the German Federal Ministry of Education and Research (BMBF). International examples are the EUROGLYCANET, targeting congenital disorders of glycosylation (CDG) since 1999 (17) and the European Rare Kidney Disease Registry (ERKReg), observing rare kidney disease since 2018 (18) both having received funding from the European Commission, or the Urea Cycle Disorders Consortium (UCDC), having received funding from the US-American National Institutes of Health (NIH) since 2003 (19). While these are notable examples, they of course represent only a small sample of the wealth of existing registries, which currently are estimated as 793 only within the scope of Orphanet (20). Therefore the landscape of rare disease registries was highly scattered when EU policy makers recognized as early as 1999, the urgency of action on rare diseases at the European level (21), starting a process that lead to the recognition of scientific patient registries as key instruments to overcome challenges specific to rare diseases, like small sample sizes, while also envisioning the introduction of European Reference Networks as a means of organizing centers of excellence (22). This resulted in rare diseases becoming an integral part of the second EU health programme, calling for funding opportunities for the development and maintenance of rare disease registries (23). In the field of inherited metabolic diseases this development allowed the founding of the European Registry and Network for Intoxication type Metabolic Diseases (E-IMD: https://www.eimd-registry.org/; CHAFEA agreement no. 2010 12 01) in 2010, the European Network and Registry for Homocystinurias and Methylation Defects (E-HOD: https://www.ehod-registry.org/; CHAFEA agreement no. 2012 12 02) in 2012, followed by the International Working Group on Neurotransmitter-Related Disorders (iNTD: https://www.intd-registry.org/) in 2014, as expert lead international patient registries (24–26). Due to the increasing number of rare inherited metabolic diseases (currently more than 1,600 diseases according to IEMbase, URL: http://www.iembase.org/), these registries were conceptualized as multi-disease registries, logically grouping diseases by common traits of clinical manifestation or affected metabolic pathways. The registries rely on a tailored custom-made IT solution, allowing secure remote entry and central storage of pseudonymized data, while enrolling patients on the protocol of an observational study, adapted to the local legal context of participating health care providers (see **Figure 1** which illustrates the modular design of electronic case report forms and study visits). The enrolment follows a top-down approach, i.e. large expert centers with great outreach in their specific countries and regions are motivated *via* their inherent scientific interest to join the consortia and enroll known and newly diagnosed patients on the various protocols. This process ensures an overall high quality of data through the expert-mediated enrolment and data entry process, leading to a good coverage for diseases whose management necessitates regular visits to large expert centers. On the other hand, this process could result in under-reporting where a more bottom-up approach mediated by patient organizations might be more beneficial. The associated health care providers organized themselves in scientific consortia, granting each member the right to use the entire wealth of gathered data for sufficiently elaborated projects, presented to and agreed by a member’s board, while at the same time guaranteeing strict individual ownership of data, thus still allowing single center research or evaluation of national cohorts, even including the right to opt out of common projects, if conflict mitigation mechanisms should fail.

The data model employed by each registry is the result of the collaborative effort of experts as well as patient organizations in the field, being perfectly suitable for capturing clinical phenotypes, but nevertheless meeting more recently established standards of semantic interoperability only to a restricted extent.

### European Rare Disease Registration Infrastructure (ERDRI) and Semantic Interoperability

**Figure 2:**
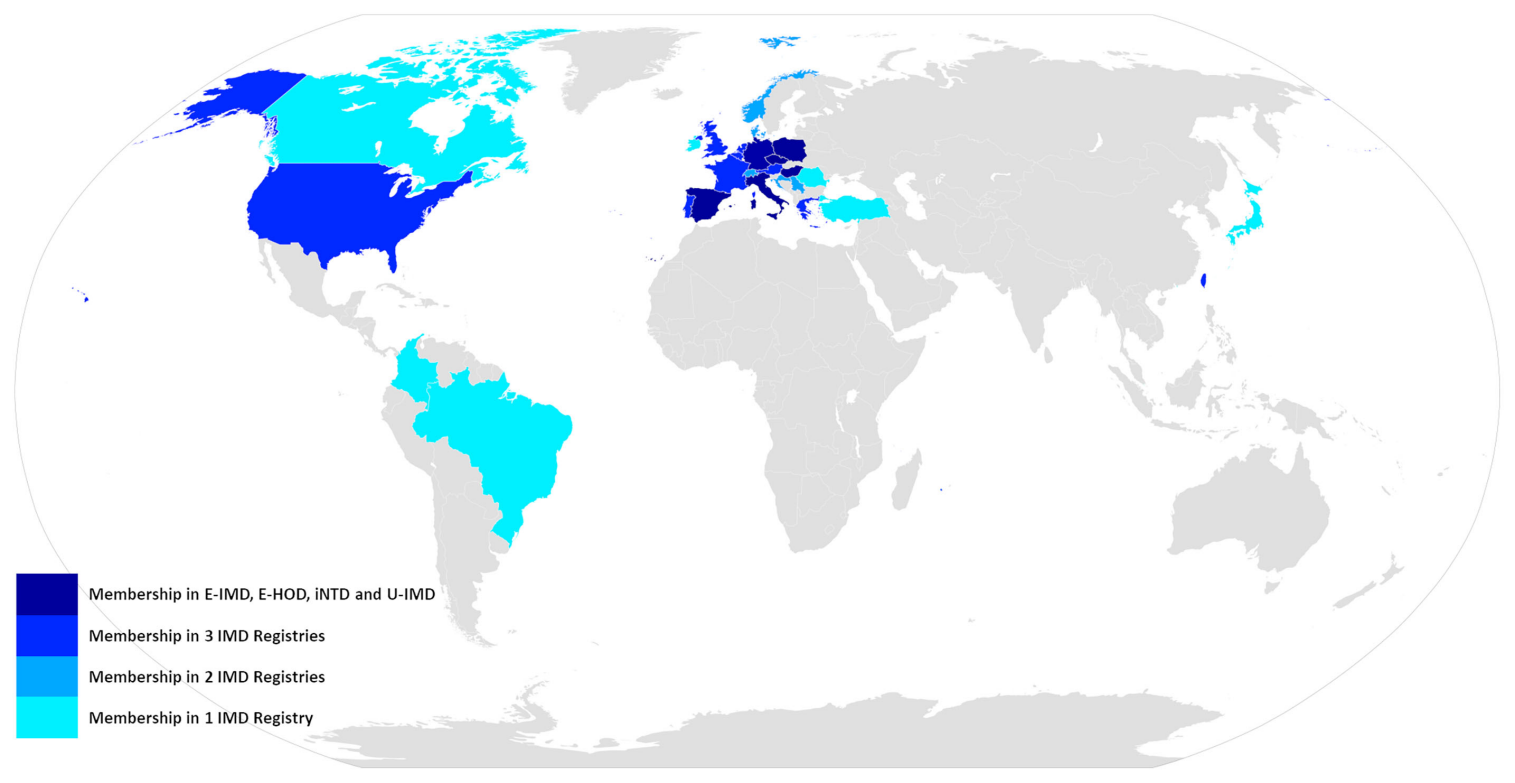
Global outreach of the E-IMD, E-HOD, iNTD and U-IMD registries.

Concomitantly to promoting individual registry building efforts the European Commission also furthered the development of the underlying framework for actions in the field of rare diseases, by establishing the advisory body of the European Union Committee of Experts on Rare Diseases (EUCERD) among others (27). The resulting EUCERD core recommendations on rare disease patient registration and data collection emphasize the need for establishing minimum common data sets, which should be uniform not only among disease specific registries but also across the entire spectrum of rare diseases, stress the critical importance of semantic interoperability between registries, which can be achieved by using standardized and controlled languages like OMIM code for case definition or SNOMED-CT for describing clinical phenotypes and endorse the introduction of a common European patient identifier (28). These principles should later materialize in the form of the European Rare Disease Registration Infrastructure (ERDRI), an integrated set of tools and recommendations presented on the European Platform for Rare Diseases. The central instrument of ERDRI is the Common Data Elements (CDE), a set of defined variables that should form the core of all European rare disease registries along with concrete recommendations on the usage of controlled dictionaries like Orpha or Alpha code for coding diseases, the nomenclature of the Human Genome Variation Society (HGVS) for describing the genetic phenotype and the Human Phenotype Ontology (HPO) for the clinical phenotype. The CDEs are embedded with further tools comprising an European metadata repository (ERDRI.mdr), an European reference database for rare disease registries (ERDRI.dor) and the introduction of an unique European patient identifier (European Platform on Rare Diseases Registration (29). With European Reference Networks having become a reality, by the time the ERDRI tools were released, the Unified Registry for Inherited Metabolic Disorders (U-IMD; CHAFEA agreement no. 777259) could be implemented as the first European and international registry encompassing all inherited metabolic diseases. U-IMD implements the ERDRI and serves as the official registry of the European Reference Network for Hereditary Metabolic Disorders (MetabERN) (see **Figure 2** for an illustration the combined global outreach of the European IMD registries and **Table 1** for the combined current enrolment numbers). Apart from introducing a new registry, the U-IMD action also aims at providing the expertise for implementing a higher level of semantic interoperability to the existing IMD registries. In this way U-IMD works as a complement and enhancement to the existing structures, avoiding duplication much as possible for a generic approach (30). The hurdles represented by lack of semantic interoperability were experienced by E-IMD in its collaborative effort to establish the largest continuously followed cohort of urea cycle disorders together with the American Urea Cycle Disorders Consortium (UCDC). Although surmountable the datasets required extensive work-up by clinicians to correctly classify clinical abnormalities since semantic interoperability of both registries was incomplete (31). Introduction of HPO terms and other controlled and structured dictionaries could have significantly shortened the mapping process (32). With these lessons in mind the adaption process facilitated by U-IMD includes the introduction of the EDRI tools to the E-IMD, E-HOD and iNTD registries, with special emphasis enhancing semantic interoperability between the registry and to other data sources with a comparable standard. U-IMD itself ensured interoperability to other ERN registries already during its design, going as far as mirroring the data model of the European Rare Kidney Disease Reference Network (ERKNet) since both ERNs share metabolic nephropathies as target diseases (30).

**Table 1:** Enrolment of active patients for the E-IMD, E-HOD, iNTD and U-IMD registries and for the German newborn screening outcome study (NGS2020, DRKS00013329).

Disease Groups according to IEM nosology	E-IMD	E-HOD	iNTD	NGS 2020	U-IMD	Total
Disorders of ammonia detoxification	570			17	124	711
Disorders of branched-chain amino acid metabolism	486				153	639
Disorders of lysine metabolism	275			10	40	325
Disorders of cobalamin metabolism	86	222		5	76	389
Disorders of sulfur amino acid and sulfide metabolism		416			54	470
Disorders of folate metabolism		72	5	1	6	84
Disorders of phenylalanine and tetrahydrobiopterin metabolism			186	169	354	709
Disorders of monoamine metabolism			136		2	138
Disorders of β- and γ-amino acids			45		1	46
Disorders of glycine metabolism			29		5	34
Disorders of serine metabolism			5			5
Disorders of fatty acid oxidation and transport				206	182	388
Disorders of branched-chain amino acid metabolism				73		73
Disorders of galactose metabolism				25	60	85
Disorders of biotin metabolism				20	46	66
Disorders of carnitine metabolism				9	25	34
Disorders of tyrosine metabolism				6	28	34
Disorders of riboflavin metabolism				4	16	20
Sphingolipidoses					208	208
Glycogen storage diseases					117	117
Disorders of cholesterol biosynthesis					72	72
Mucopolysaccharidoses					66	66
Disorders of mitochondrial tRNA					48	48
Disorders of lysosomal cholesterol metabolism					43	43
Disorders of gluconeogenesis					40	40
MITOCHONDRIAL DISORDERS OF ENERGY METABOLISM					109	109
DISORDERS OF CARBOHYDRATES					64	64
DISORDERS OF NITROGEN-CONTAINING COMPOUNDS					58	58
CONGENITAL DISORDERS OF GLYCOSYLATION					39	39
DISORDERS OF PEROXISOMES AND OXALATE					33	33
DISORDERS OF LIPIDS					30	30
STORAGE DISORDERS					21	21
DISORDERS OF VITAMINS, COFACTORS AND MINERALS					11	11
DISORDERS OF TETRAPYRROLES					2	2
**Total**	**1417**	**710**	**407**	**545**	**2133**	**5212**

All patient numbers as of 30^th^ November 2021.

## Patient Registries Are Multi-Purpose Instruments for Rare Disease Research

### Natural History Studies

The most common application for patient registries is natural history studies, e.g., ultra-rare diseases such as mevalonic aciduria (33), to raise awareness. The precise description of the initial presentation helps to reduce diagnostic delay and to guide the diagnostic process. Noteworthy, phenotypic diversity can be extreme such as in urea cycle disorders, ranging from life-threatening sepsis-like hyperammonemic encephalopathy in newborns with severe disease onset to variable fluctuating episodes of neurological, gastrointestinal and psychiatric symptoms in patients with attenuated forms (34, 35). Phenotypic diversity of variant disease courses can be easily mistaken as discrepant diseases with overlapping biochemical and clinical presentations. This is exemplified by genotype phenotype correlation studies in neuroblastoma-amplified sequence (NBAS)-associated disease which identified three different but partially overlapping subgroups with complex phenotypes which are directly related to the affected region of the NBAS protein (36). In order to distinguish the initial presentation and the evolving clinical phenotypes of rare diseases, comparative phenotyping within and between disease groups is a helpful strategy. Recently, this approach was successfully applied to elucidate clinical similarities and differences between disorders of biogenic amine and tetrahydrobiopterin (BH_4_) metabolism (37), urea cycle disorders, and organic acidurias (24). This knowledge is of relevance for the optimization of the diagnostic process, patient clinical paths, healthcare planning, and counselling of parents having a child with a rare disease. For example, comparative phenotyping helped to identify the need to carefully monitor renal function not only in individuals with isolated methylmalonic acidurias but also in individuals with other organic acidurias such as propionic aciduria and glutaric aciduria type 1 (24, 38) since chronic kidney dysfunction was already well known in methylmalonic aciduria (39, 40), but was underestimated in propionic aciduria and unknown in glutaric aciduria type 1 before. Patient registries also allow comparison between different diagnostic journeys and related health outcomes on an international level, especially if there are known rigid differences in diagnostic approaches between countries, that may be evaluated against each other, as is the case with differing national newborn screening panels. Results from such comparisons may help in harmonizing newborn screening programs by illustrating different needs for long term care resulting from different diagnostic journeys in the light of health economics.

Patient registries are powerful tools to describe and compare disease variants and to challenge existing case definition, disease classification, and indication to treat. Recently, careful biochemical and clinical evaluation of more than 300 individuals with confirmed cystathionine β-synthase deficiency resulted in the development of comprehensive criteria for the classification of pyridoxine responsiveness (41). This reclassification is likely to have enormous practical consequences on the diagnostic process, treatment and management of affected individuals with this disease since pyridoxine responsiveness is the major predictor of clinical severity and outcome. Furthermore, patient registries can help to elucidate the clinical relevance of newly identified disease variants with yet uncertain clinical significance. A good example is so-called mild isovaleric aciduria, an attenuated and putatively benign disease variant, which has been increasingly recognized since the inclusion of isovaleric aciduria into newborn screening panels. In contrast to individuals with the severe, so-called classic, disease variant with high risk of neonatal mortality, poor neurocognitive outcome in surviving individuals, and a clear indication to treat, individuals with the mild disease variant are not confronted with acute metabolic decompensations and increased neonatal mortality and showed excellent neurocognitive outcomes. Noteworthy, excellent health outcomes in individuals with mild isovaleric aciduria did not appear to be impacted by metabolic maintenance therapy (42), questioning the overall benefit from NBS and the indication to treat.

A solid description of the disease course with its age-dependent manifestations and variations is also an important source for the identification of meaningful endpoints for clinical trials instead of surrogate parameters with doubtful clinical relevance for affected individuals. Furthermore, it can guide the inclusion of individuals with well-defined clinical severity to clinical trials in order to avoid apparent (non-) effectiveness of the study drug due to case mix differences of study groups. For individuals with urea cycle disorders, the severity of the initial presentation, represented by age at disease onset, initial peak ammonium concentration in plasma, and coma, has a major impact on survival and neurocognitive outcomes in urea cycle disorders (43, 44). In addition to mortality as a hard clinical end point, collaborative evaluation of 300 individuals with ornithine transcarbamylase deficiency identified intelligence as another suitable clinical endpoint since it was shown to be the most distinguished indicator for cognitive function and to correlate highly with the initial peak ammonium concentration in plasma (45, 46).

### Benefits and Limitations of Current Diagnostic and Therapeutic Strategies

It is a well-known fact that individuals with rare diseases are often confronted with a diagnostic odyssey and diagnostic delay, putting high burden on the affected individual and her/his family and being associated with an increased risk of losing therapeutic effectiveness and developing irreversible organ dysfunction. To shorten the path to diagnosis newborn screening programs for rare diseases, such as inherited metabolic diseases, have been continuously developed for more than 50 years following Robert Guthrie’s pioneering work (47). With technological developments, such as tandem mass spectrometry, and concomitant extensions of the disease panel, endogenous intoxication-type metabolic diseases with acute neonatal manifestations have been introduced to these programs (48, 49). As a consequence, some individuals affected by these diseases may become symptomatic in the first days of life, even before newborn screening results are available, questioning the benefit of newborn screening. Comparison of screened and unscreened cohorts followed by patient registries can help to estimate the proportion of individuals who can be reliably identified pre-symptomatically depending on the time point of sample collection (44, 50). Such studies can also evaluate the diagnostic process quality of newborn screening programs, identifying current strengths and weaknesses of its team-players, i.e. senders, mail services, and laboratories, evaluate promising new biomarkers, such as 3-O-methyldopa in dried blood spots for aromatic ʟ-amino-acid decarboxylase deficiency, and thus can highlight the potential for improvement (33, 51). Longitudinal outcome studies of newborn screening cohorts, which provide formal evidence of the clinical effectiveness and the long-term health benefits of screened individuals, have remained the neglected part of newborn screening programs. A long-term observational study in Germany recently demonstrated that newborn screening for inherited metabolic diseases resulted in overall normal development (96%), normal cognitive outcome (mean IQ: 100), and regular attendance of screened individuals at kindergarten (95%) and primary school (95%) (51). At the same time, it identified disease-specific variations of health outcomes and diseases with less favorable health outcomes, particularly if recommended treatment was not adequately prescribed or adhered to (38, 52), as well as individuals with a putatively benign disease variants with risk of over-treatment (42). In addition, observational studies based on patient registries are helpful to evaluate the prevalence and putative health impact of early diagnosis and treatment for conditions that are not yet regularly included in national screening panels, such as neonatal vitamin B_12_ deficiency mostly due to hitherto undiagnosed maternal vitamin B_12_ deficiency (53).

Another important but sometimes difficult application of patient registries is the evaluation of therapeutic safety and effectiveness under real-world conditions. In contrast to clinical trials, inclusion and exclusion criteria are kept to a minimum in patient registries in order to study affected individuals with a broad phenotypic spectrum (1). Furthermore, patients are observed as they present for care instead of asking for strict adherence to protocol. Because of the resulting high variation, which is important to achieve generalizable results, evidence-based answers to therapy-related questions often depend on a large sample size and hence require the pooling of data from different sources (44, 46). Interoperability is key to the success of this effort. If these hurdles can be overcome, patient registries are valuable sources for the optimization of therapies and guideline development. For instance, long-term follow-up of the largest cohort of individuals with urea cycle disorders did not find evidence for superiority of any of the available nitrogen scavenger therapies but highlighted that early liver transplantation appears to be beneficial (46). Another example is glutaric aciduria type 1, which was thought to be an untreatable condition in the pre-screening era since treatment given to symptomatic patients did not improve the clinical outcome (54). Introduction of tandem mass spectrometry-based newborn screening programs challenged this view, proving that pre-symptomatic start of treatment is the major prerequisite for favorable neurological outcome (38, 52). Long-term follow-up of two large patient cohorts in the USA and Germany further elucidated that in screened individuals with glutaric aciduria type 1 therapeutic quality and adherence to evidence-based recommendations become major predictors of neurological outcome and survival (55). Furthermore, these studies showed that low lysine diet supplemented with fortified metabolic formula is superior to protein restriction and allows for normal anthropometric long-term development (38, 56, 57). In another large group of individuals with protein-dependent inherited metabolic disorders, study of the dietary impact on growth highlighted the need for careful monitoring of plasma amino acids, particularly branched-chain amino acids and L-arginine, and to adjust the protein-to-energy prescription of diet to support normal growth (58). In brief, besides precise evaluation of the natural history patient registries are also valuable instruments to assess the iatrogenic impact on the evolving clinical phenotype.

### Guideline Development

Patient registries are important data sources for guideline development, helping to improve the evidence level and the grade of recommendation. Since guideline development for rare diseases is often difficult and based on literature with a low level of evidence, such as case reports, small case control or cohort studies, all of them having a high risk of bias and low probability that the reported causes and effects are causally linked. Furthermore, guideline development often identifies topics which have remained white spots on the scientific map, with no published evidence at all. Observational studies and health economic evaluations based on patient registries can specifically address these questions and topics to larger cohorts, seeking for evidence. Furthermore, patient registries can inform guideline developmental groups about the feasibility, safety, and effectiveness of guideline recommendations, paving the way for successful guideline revision. The high potential of this approach can be illustrated by guideline development for glutaric aciduria type 1, being initiated more than 15 years ago. While the first guideline mostly included grade D and a few grade C recommendations, reflecting significant uncertainty (59), the level of evidence and grade of recommendations have been improved continuously, demonstrated by the first (60) and second revision (61). This progress was much accelerated by careful long-term follow-up of national and international cohorts (38, 56, 57, 59, 61–69), demonstrating that (i) newborn screening is the prerequisite of favorable neurological outcome and survival and (ii) is highly cost-effective, and that (iii) adherence to recommended maintenance and emergency treatment is associated with the most favorable neurological outcome and (iv) normal growth. Furthermore, these studies described a so far unknown renal disease manifestation, unraveled similarities (risk of striatal necrosis with concomitant complex movement disorder with predominant dystonia) and discrepancies (cognitive function, white matter changes, subdural hematoma) between biochemically delineated subgroups (high versus low excreter phenotype), and evaluated which part of the complex clinical spectrum can be specifically targeted and changed by current therapy, highlighting the need for safer and more effective medicines. Similar approaches to continuous improvement of guidelines for rare diseases through long-term observational studies coordinated by international scientific consortia have also been chosen for other rare diseases, such as urea cycle disorders (70, 71), propionic and methylmalonic aciduria (72, 73), cobalamin-related remethylation disorders (74–76), cystathionine beta-synthase deficiency (41, 77), and neurotransmitter-related disorders (37, 78, 79).

### Post-Authorization Safety Studies (PASS)

Large scale data collection whether in clinical research or other scientific fields, raises concerns regarding the principles of long-term data stewardship, having resulted in the formulation of the FAIR concept, signifying findability, accessibility, interoperability and reusability of data (80). The aspect of reutilization has a special ethical significance in clinical research for rare disease, since comparatively small patient cohorts imply an enhanced risk of putting the burden of participation in multiple studies on a small number of patients, often severely affected. For the same reason of small patient numbers, orphan drug designations and the resulting need for post-authorization safety studies (PASS) are common for rare diseases, which usually are implemented as industry-driven single purpose drug registries. There is an increasing awareness of regulating bodies like the European Medicine Agency (EMA) for the under-utilization of existing data sources like scientific disease registries, which often already gather the type of longitudinal data also required for certain regulatory actions like post-authorization measures (81, 82). Therefore, the collaboration between marketing authorization holders (MAH) and scientific patient registries, within the model of a public private partnership, is a feasible approach for achieving the goals of certain drug safety measures, while at the same time reducing data fragmentation and duplication and ensuring ongoing data stewardship by publicly funded scientific consortia, better capable and more committed to ensuring data FAIR-ness than industry lead efforts. In the past E-HOD, as well as currently E-IMD, successfully implemented the model as envisioned by the EMA, for the orphan drugs Cystadane^®^ (betaine anhydrous, http://www.encepp.eu/encepp/viewResource.htm?id=40022) and Ravicti^®^ (glycerol phenylbutyrate, EUPAS17267; URL: http://www.encepp.eu/encepp/viewResource.htm?id=30377), respectively. In both cases the PASS studies were implemented on a protocol drafted by the MAH together with the scientific registries and accepted by the Pharmacovigilance Risk Assessment Committee (PRAC) of the EMA. Patients already being enrolled in the respective registries could choose to also participate in the PASS, allowing their already available, purely observational data to be used for facilitating the surveillance measure, without increasing their burden or generating new data that would be only available in an industry owned data source.

## Discussion

Patient registries are key to personalized evidence-based medicine for individuals with rare diseases since they help to overcome the intrinsic obstacles of rare disease research through pooling of data and achievement of sufficient sample sizes. There is an increasing number of examples demonstrating that patient registries can fulfil multiple purposes for rare disease research, particularly (A) improving the knowledge about natural history and variant disease courses, (B) identifying meaningful endpoints for clinical trials, improving case definition, genotype phenotype correlation, risk stratification, and therapeutic decision, (C) evaluating the safety, effectiveness, and long-term health benefits as well as the health economical and societal benefits of preventive care programs, diagnostic strategies, and therapies, such as orphan drugs, and (D) increasing the evidence base and recommendation grade of guidelines.

However, successful establishment, sustainability, and usefulness of patient registries may be limited by (A) data fragmentation and duplication because of uncoordinated parallel activities with unharmonized data models, (B) lack of seeding and sustainability funding though industry-independent public sources, hampering projects *via* numerous funding dependent constraints like reimbursement of person hours invested by experts, (C) insufficient geographical coverage resulting in small cohorts, (D) non-adherence to data FAIRness, particularly syntactic and semantic interoperability, hampering data exchange and cross-site analysis, (E) non-compliance with data protection regulations resulting in the discontinuation of some older patient registries following the inception of the GDPR in 2018, and (F) non-involvement of patients and patient groups in the development of registries, leading to insufficient consideration of the patients’ view and experience. This last point is of special significance, since conservative treatment options for many IMDs can put a high psychosocial burden on patients and caregivers alike, often necessitating measures like permanent strict dietary management that conflict with many life choices. Adequately capturing these burdens with tailored patient reported outcome measures (PROMs) was often neglected in the past, but will become increasingly important going forward, since novel treatments directly addressing underlying disease causes will increasingly become available, with PROMs being an important measure for their efficacy (83).

In conclusion, the future success of patient registries for rare disease research in Europe critically depends on the compliance with formal requirements, particularly the FAIR data principles and the GDPR to ensure long-term data collection and data exchange in a protected environment. As indicated by a recent survey among 40 rare disease registries, there seems to be a high level of recognition within the community that adherence to formal aspects of governance, particularly FAIR principles, is a key criterion of successful implementation and administration of rare disease registries (84), although especially long-running projects might struggle to keep pace with adapting to recently introduced standards. Particular attention should be paid to facilitating the achievement of ethical approval and informed consent since these hurdles are importing rate-limiting steps (19, 24, 30). With the future development of safe and reliable IT tools for data extraction from electronic health records it is also hoped that the work- and cost-intensive process of gathering and entering personalized health data to registries can be significantly reduced. Finally, industry-independent sustainability of patient registries conducted by scientific consortia and European Reference Networks for Rare Diseases requires the establishment of long-term funding strategies on the level of the European Union and its Member States.

## Author Contributions

All authors conceptualized the manuscript. SK and FG wrote the first draft of the manuscript. UM and TO edited the manuscript. All authors contributed to the article and approved the submitted version.

## Funding

Discussed studies, coordinated by some of the authors, received funding: SK received EU funding for the European Registry and Network for Intoxication-type Metabolic Diseases (E-IMD; CHAFEA agreement no. 2010 12 01), the European Network and Registry for Homocystinurias and Methylation Defects (E-HOD; CHAFEA agreement no. 2012 12 02), and the Unified Registry for Inherited Metabolic Diseases (U-IMD, CHAFEA agreement no. 777259), as well as funding from the Dietmar Hopp Foundation (Germany) for the observational study NGS 2020. TO received funding by Dietmar Hopp Foundation for conducting the International Working Group on Neurotransmitter-Related Disorders (iNTD).

## Conflict of Interest

The authors declare that the research was conducted in the absence of any commercial or financial relationships that could be construed as a potential conflict of interest.

## Publisher’s Note

All claims expressed in this article are solely those of the authors and do not necessarily represent those of their affiliated organizations, or those of the publisher, the editors and the reviewers. Any product that may be evaluated in this article, or claim that may be made by its manufacturer, is not guaranteed or endorsed by the publisher.
